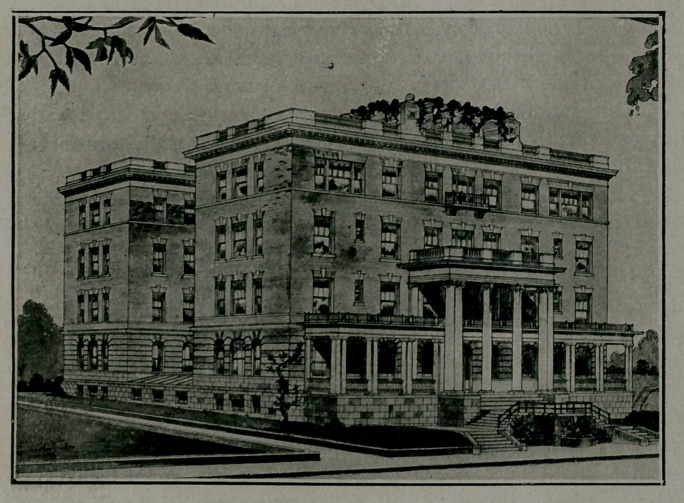# Editorial

**Published:** 1912-04

**Authors:** 


### EDITORIAL

The Business Office of the *Journal-Record* is 1 1-2 to 5 1-2 South Broad Street.
The Editorial Office is 1014-15 Century Building.
Address all Business Communications to *Journal-Record of Medicine*, 1 1-2 to 5 1-[illegible] South Broad Street.
Make remittances payable *to The Journal-Record of Medicine.*

On matters pertaining to the Editorial and Original Communications, address Edgar G. Ballenger, M. D., Atlanta, Ga.
Reprints of Original Articles will be furnished at cost price. Requests for the same should always be made in THE MANUSCRIPT.

We will present, postpaid, on request, to each contributor of an original article, twenty (20) marked copies of *The Journal-Record of Medicine* containing such article.

### DR. AMSTER LEAVES FOR EUROPE.

Dr. Amster left for Berlin on the S. S. Kaiser Wilhelm der Grosse, complying with the urgent request of Dr. Hans Elsner, for many years first assistant of Professor Boas. Dr. Amster will assist Dr. Elsner in the Boas-Elsner Polyclinic.

Professor Boas has retired from the Polyclinic and discontinued his lectures and has installed Dr. Elsner in his stead.

There are many English and American physicians taking courses at this Polyclinic and Dr. Elsner desiring to give his lectures in the English language has invited Dr. Amster to join him. While in Berlin Dr. Amster will devote himself to other work and has promised to furnish the Atlanta Journal-Record of Medicine with an occasional contribution.

### SUBSTANTIAL ACKNOWLEDGEMENT OF CRAWFORD W. LONG'S PRIORITY IN THE USE OF ETHER AS AN ANAESTHETIC.

The following invitation received by the editor of the Journal-Record of Medicine is welcomed with pleasure, as Long's discovery of ether as an anaesthetic was so long a disputed question. This substantial acknowledgement by such a well-known historic institution will permanently place Long's name among the "immortals" in medicine, where it justly belongs:

The Provost and Trustees of the University of Pennslyvania request the honor of your presence in the hall of the Medical Laboratory Building 37th and Hamilton Walk, at 2 P. M., Saturday, March 30th, 1912, on the occasion of the unveiling of a bronze medallion in honor of Dr. Crawford Williamson Long, 1839, Med. U. and P., who first made use of ether as an anaesthetic for surgical purposes, on March 30th, 1842. Addresses by Dr. J. William White and Dr. J. Chalmers Da Costa. Music by the University Glee Club.

---

### A MODERN SANITORIUM.

A little more than two years ago, Drs. E. C. Davis and L. C. Fischer leased a building on Crew St. for a Sanatorium and for two years conducted this according to their ideas.

So successful did this prove that they were induced to purchase a lot on Linden Avenue between Peachtree and West Peachtree

Streets and to erect a modern fire-proof building with one hundred rooms and all the new ideas of sanatorium construction, including open air spaces, roof garden covering whole building with sleeping quarters, wide porches, sanitary plumbing, signal lights instead of bells and all the appurtenances that go with the strictly advanced sanatorium of to-day.

This has been constructed sufficiently large to accommodate the patients of reputable physicians who desire to avail themselves of these advantages.

This building has placed within this section a thoroughly fire-proof hospital where the sick can feel perfectly secure, and the diet arrangements completed so that food may be served attractive, thoroughly equipped to accomplish these purposes.

Such an institution has been long needed in this section and the profession, as well as the sick, will appreciate the privileges offered. So many sanatoriums and hospitals have been built without a thought of their inflammable possibilities and the poor, suffering patients, in case of fire, would be placed in a most pitiable position. Then the comforts can not be obtained usually in remodeled residences and antiquated homes such as are often converted into hospitals and sanatoriums.

This building has been constructed for a Sanatorium and has embodied all the newer ideas pertaining to the comforts of patients and thoroughness of results.

The Surgical Section with two operating rooms, one for septic and minor cases and the other for major, should greatly reduce the possibilities of infection. The Operating Rooms are especially well arranged, tiled to the ceiling with double glass, electric ventilators and are easily flushed. In fact, this Sanatorium offers advantages surpassed by none in the South.

---

So gratifying is it to see at last the recognition of Dr. Crawford W. Long as the first to use ether as an anaesthetic that we devote much space in this issue to an article which was recently published in the Lancet, of London, and which we know will be of interest to all Southern and especially Georgia physicians and surgeons.

## Figures and Tables

**Figure f1:**